# Bone Marrow Mononuclear Cells and Plasma Gel as Combination Treatment for Hard-to-Heal Wounds

**DOI:** 10.3390/life16050847

**Published:** 2026-05-20

**Authors:** Silvia Perez-Lopez, Nuria Vazquez-Garcia, Maria Luz Rodriguez-Martinez, Susana Valerdiz-Casasola, Marcos Perez-Basterrechea, Jose Maria Garcia-Gala, Maria de los Angeles Fernandez-Rodriguez, Eva Martinez-Revuelta, Maria Alvarez-Viejo

**Affiliations:** 1 Unidad de Terapia Celular y Medicina Regenerativa, Servicio de Hematología y Hemoterapia, Hospital Universitario Central de Asturias, 33011 Oviedo, Spain; 2 Instituto de Investigación Sanitaria del Principado de Asturias, The Fundación para la Investigación e Innovación Biosanitaria en el Principado de Asturias (FINBA), 33011 Oviedo, Spain; 3 Unidad de Heridas Crónicas, Hospital Universitario Central de Asturias, 33011 Oviedo, Spain; 4 Área de Psicobiología, Departamento de Psicología, Universidad de Oviedo, 33003 Oviedo, Spain

**Keywords:** wounds, tissue engineering, bone marrow mononuclear cells, plasma scaffold

## Abstract

Hard-to-heal wounds pose a significant challenge in clinical practice due to the fact that the conventional treatments used are not always effective. For this reason, it is necessary to design alternatives to achieve an adequate resolution. In this context, a new Advanced Therapy product was produced in a Good Manufactured Practices Facility in the setting of a clinical trial authorised for the European Medicines Agency (EUCT 2023-505017-25-02). Briefly, an autologous plasma scaffold containing bone marrow mononuclear cells was applied to a 63-year-old male patient who presented a non-healing wound despite two months of self-care and three months of primary care treatment. After cleaning the affected area, a single-dose plasma scaffold with embedded bone marrow mononuclear cells was applied over the wound. Six weeks after treatment, the wound exhibited remarkable healing with complete closure as evidenced by follow-up assessments at different time points. Quality of life measures significantly improved, aligning with clinical findings, and no adverse effects were observed. While further studies are needed, the issues presented in this case report show the promising results obtained forthe first patient included in the trial and treated with this innovative alternative, which supports the potential of mononuclear cells combined with plasma as a therapeutic option for chronic wounds.

## 1. Introduction

The term skin wound refers to any disruption in the continuity of the skin following trauma or tissue stress [1]. When a skin wound occurs, the body initiates a well-orchestrated healing process designed to regenerate the injured area, a process that involves a cascade of biological mechanisms including inflammation, proliferation, and remodelling. Under optimal conditions, these sequential phases operate efficiently to restore tissue integrity. However, this process can be compromised by both systemic and local factors such as advanced age, vascular insufficiency, malnutrition, or uncontrolled diabetes, which may alter oxygenation, immune response, or cellular function and ultimately lead to chronic wounds [2]. Chronic injuries, also known as hard-to-heal wounds or chronic ulcers, are defined as those that do not heal properly over a normally adequate period of time—typically failing to reduce in size or progress through normal physiological stages within three months [3]. These wounds not only affect the quality of life of the people who suffer from them, limiting mobility and autonomy and generating pain or recurrent infections, but also represent a major concern for public health systems throughout the world due to their high prevalence and the significant costs associated with their long-term management [4]. While it is simple to define a chronic wound from a conceptual standpoint, finding an effective solution in daily clinical practice is often challenging [5]. Conventional treatment typically relies on infection control, adequate wound bed preparation through periodic debridement, moisture balance via appropriate dressings, and, when needed, surgical interventions such as skin grafts or flap coverage [6]. Unfortunately, these approaches do not always lead to successful outcomes, particularly in patients with comorbidities or compromised vascularization. It is therefore necessary to develop new therapeutic strategies that can enhance the healing of these chronic wounds and overcome limitations seen with standard care. In this context, tissue engineering has emerged as a promising and innovative option. Tissue engineering is a multidisciplinary field that combines expertise in cell biology, materials science, engineering, and clinical research with the aim of creating tissues or organs capable of repairing or replacing those that have been damaged [7]. The engineering process usually relies on two fundamental elements: a structure or scaffold, often three-dimensional, which mimics the extracellular matrix, and living cells that populate and interact with this structure. Among the available scaffold options, human-derived biological materials such as blood components can be particularly advantageous. The benefits of these autologous constructs include excellent biocompatibility, inherent biodegradability, capacity for cell adhesion, low immunogenicity, and minimal toxicity. Bone marrow mononuclear cells (BM-MNCs) are a heterogeneous cell population that has been used for several years in the field of regenerative medicine, demonstrating encouraging results in the treatment of various conditions including ischemic diseases, long bone pseudoarthroses, and pressure injuries. Our unit has developed a procedure for the Good Manufacturing Practice (GMP) production of autologous BM-MNCs in saline as an Advanced Therapy Medicinal Product (ATMP). This product has been used in several approved clinical trials to treat pressure injuries and long bone pseudoarthroses with positive outcomes [8,9]. However, administering these cells as a suspension in saline may not be the most appropriate formulation for treating certain conditions such as hard-to-heal wounds, where prolonged contact with the wound bed and a supportive matrix could enhance therapeutic efficacy.

An autologous plasma gel could serve as an ideal scaffold, providing both structural and mechanical support to BM-MNCs and facilitating their sustained interaction with the damaged tissue. In an initial preclinical stage of research focusing on a mouse model of pressure injury, we observed that healing was significantly accelerated when non-expanded BM cells were embedded within a plasma gel scaffold, compared to control conditions [10]. These results supported the potential of this combined therapeutic approach and led to the granting of EMA authorisation for a clinical trial designed to assess this new ATMP (BM-MNCs embedded in autologous plasma gel) for the treatment of hard-to-heal wounds (EUCT 2023-505017-25-02). Herein, we report the first use of this treatment in a patient.

## 2. Clinical Case

A 63-year-old man was referred to the Chronic Wound Care Unit of the Central University Hospital of Asturias, Oviedo, Spain. The patient presented a wound area of 7.06 cm^2^ over the Achilles tendon of the right leg due to a traumatic cause provoked by continuous rubbing and friction in an area of skin fragility due to a very prominent tendon and frequent use of motorcycling boots. After vascular examination of the right lower limb, no oedema was observed; skin colour and temperature were normal, sensation was intact, pulses were present, and the ABI (ankle–brachial index) was within the normal range. Upon referral from primary care to the Chronic Wound Unit at the Hospital Universitario Central de Asturias, the patient had failed to respond to three months of self-care and two months of standard topical treatment in a primary care setting which included a hydrofibre dressing (Aquacel Extra). The patient’s medical history included hypertension and hyperuricemia but no history of diabetes, cardiovascular disease, prior surgeries, trauma, cancer, or neoplasia (the latter being exclusion criteria for trial participation). Physical examination revealed a body temperature of 36.4 °C, blood pressure of 185/98 mmHg, and a heart rate of 71 bpm. No other chronic conditions were identified. Once the patient was admitted to the Chronic Wound Unit, two months before Advanced Therapy treatment, therapeutic management followed a step-up approach for three weeks—initially utilising Negative Pressure Wound Therapy (NPWT) at −80 mmHg to promote angiogenesis and later supplemented with a 3D collagen dressing CutimedEpiona (Essity, Tarragona, Spain) to modulate metalloproteinases. Once these preliminary treatments were completed and after clinical stagnation for more than a month, the patient was transitioned to a cellular therapy intervention, prioritised for its regenerative potential. Baseline laboratory tests, including a complete serological study, were completed following the initial consultation and deemed suitable. After reviewing the results, the patient provided informed consent, and treatment with the ATMP was scheduled.

## 3. Materials and Methods

**Figure 1:**
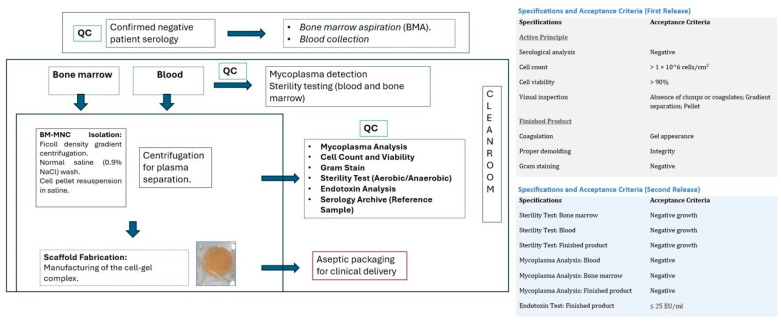
The scheme provides a summarised view of the process and indicates, with “QC,” the quality control points carried out throughout the entire process. On the right, a table presents the specifications and the criteria that must be met for both the first and second product release.

The manufacturing process was conducted in a Grade A clean room environment following standardised protocols. Before the processing of the starting materials (bone marrow and peripheral blood), samples were collected for in-process mycoplasma and sterility testing. To produce the ATMP, approximately 18 mL of the patient’s whole blood were collected in sodium citrate-containing tubes (BD, New Jersey, NJ, USA). In parallel, 15 mL of bone marrow were obtained from the posterior–superior iliac crest under topical anaesthesia (Mepivacaine 2%, B. Braun, Melsungen, Germany). In the GMP facility, whole blood was centrifuged at 1300× *g* for 20 min at 20 °C, after which plasma was collected and kept at room temperature for scaffold preparation. Bone marrow mononuclear cells were isolated using Ficoll-Paque™ PREMIUM density gradient centrifugation (1.077 g/mL; Cytiva, Marlborough, MA, USA) at 400× *g* for 25 min at 20 °C, washed in saline (0.9%; Grifols, Barcelona, Spain), and counted. The plasma scaffold was prepared in a 6-well tissue culture plate (Avantor, Radnor, PA, USA) by mixing 3 mL of plasma with one million cells per cm^2^ and adding 60 μL of tranexamic acid (Amchafibrin 100 mg/mL, Viatris, Pittsburgh, PA, USA). Coagulation was induced by adding 300 μL of CaCl_2_ (B. Braun, Melsungen, Germany), and the plate was incubated at 37 °C with 5% CO_2_ for 40 min to allow for complete gelification. The resulting scaffold was carefully removed and placed on Linitul^®^ (Alfasigma, Bolonia, Italia), previously cleared of its oily layer. To ensure product safety and reproducibility, two identical scaffolds were fabricated: one for clinical application and a duplicate for quality control (QC) analysis. The primary release criteria included assessment of cell count (>1 × 10^6^ cells/cm^2^), viability (>90%), Gram staining (negative), and macroscopic gel consistency. A secondary release confirmed the absence of endotoxins, mycoplasma, and long-term microbial growth. Reference samples were stored in a serolibrary for retrospective monitoring. A schematic representation of the complete process is provided in Figure 1, indicating the quality control (QC) checkpoints. This figure also summarises the parameters required for product release. The scaffold used in this study met all the established criteria for both the first and second release. The scaffold was transferred in saline to the treatment room. After wound cleaning, the single-dose plasma scaffold with embedded BM-MNCs was applied directly over the ulcer and covered with a polyurethane foam dressing Aquacel^®^ Foam Pro (Convatec, London, UK). Wound care at 5 days and at 2, 4, and 6 weeks included cleaning with 1% aqueous chlorhexidine (Cristalmina, Salvat, Barcelona, Spain), application of hydrocolloid hydrofibre (Aquacel^®^ Extra™, Convatec), and coverage with Aquacel^®^ Foam Pro.

## 4. Results

**Figure 2:**
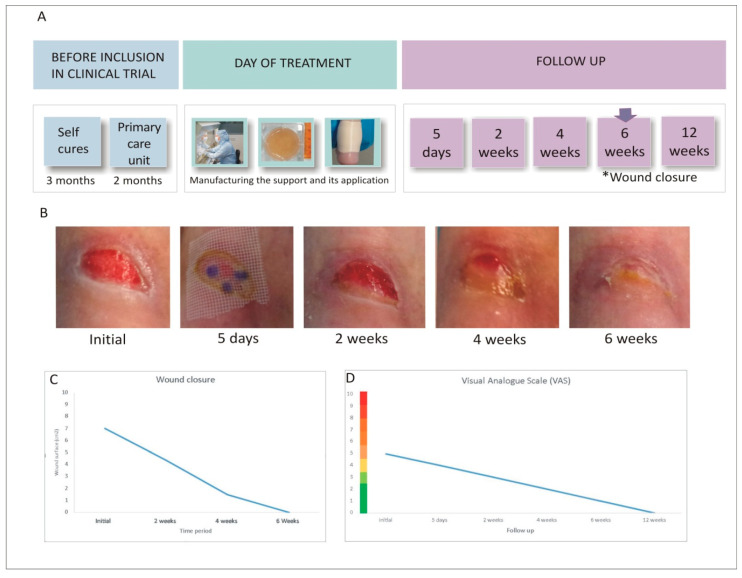
(**A**) Steps of the treatment intervention. (*) indicates wound closure 6 weeks after treatment (**B**) Images of the lesion taken during the different visits showing the ulcer healing process in response to treatment. (**C**) Wound area measured at different time points. (**D**) Trend recorded in pain assessed using the VAS scale.

Wound closure was evaluated through dimensional measurements (Figure 2B,C) and considered complete when fully covered by epithelial tissue. Clinical progression was monitored using the RESVECH 2.0 scale, and wound area was calculated with the ellipse formula (a × b × π). Pain was assessed using the visual analogue scale (VAS), and the influence of treatment on daily functioning was evaluated using the EuroQol quality of life questionnaire (EQ-5D). A schematic of the full intervention is provided in Figure 2A.

**Figure 3:**
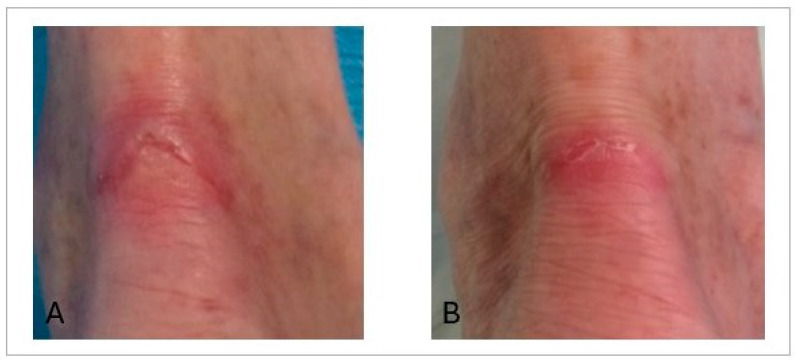
Clinical appearance of the wound area at the 6-month follow-up and (**A**) at the 12-month follow-up (**B**). Complete epithelialization and tissue stability are observed following BM-MNC therapy, with no signs of recurrence.

The clinical effects of ATMP treatment were favourable. The initial wound area of 7.06 cm^2^ steadily decreased to 4.39 cm^2^ at week 2 and 1.50 cm^2^ at week 4. By week 6, the wound area reached zero, indicating complete closure (Figure 2C). Wound dimensions were assessed manually using a standardised ruler technique. To ensure consistency, a single investigator performed all measurements. Although we are aware of the inherent limitations of the elliptical formula compared to digital planimetry, particularly regarding the potential for area overestimation, the longitudinal consistency of the measurement process allowed for a reliable assessment of the clinical healing trend. The RESVECH 2.0 score improved from 8 at baseline to 5 after 4 weeks, and further to 1 at three months. The EuroQol (EQ-5D) score increased from 80 before treatment to 95 at week 4 and 98 at three months. Pain, measured using the VAS scale, decreased markedly from 5/10 to 1/10 after six weeks (Figure 2D). Long-term evolution was assessed during 6- and 12-month follow-up visits. In both instances, complete wound closure was maintained without signs of recurrence, as shown in Figure 3.

## 5. Discussion and Conclusions

Considering the global burden of hard-to-heal-wounds, which are responsible for serious clinical complications and impose high financial costs on healthcare systems, as studied by Ongarora et al. [11], exploring alternative therapeutic approaches is needed to improve this situation. In this type of setting, advanced cell therapies could be a suitable treatment option. To treat chronic wounds, different types of scaffolds have been combined with cells or cell products based on the specific properties of each such as biocompatibility or angiogenic potential. In this regard, Wang et al. [12] combined a hydrogel with adipose-derived mesenchymal stem cell (MSC) extracellular vesicles to treat chronic diabetic wounds. Moon et al. [13] completed a multicentre clinical trial in 2019 to assess the use of a hydrogel-based allogeneic adipose-derived stem cell (ASC) sheet for the treatment of diabetic foot ulcers. In a recent study, Zhou et al. [14] obtained improved results over those achieved with conventional treatment with peripheral blood mononuclear cells by combining umbilical Wharton’s jelly-derived MSCs with platelet rich plasma to expedite wound healing in a porcine wound model. Research published by Rustad’s group in 2012 explored the efficacy of pullulan–collagen hydrogel constructs in delivering MSCs to wound sites. While systemic or local MSC injections did not yield a significant improvement in closure rates, the hydrogel-based delivery system significantly enhanced the healing process. Rustad et al. observed wounds treated with MSC-seeded hydrogels, which demonstrated significantly enhanced angiogenesis, associatedwith increased levels of VEGF and other cytokines within the wounds. The authors suggest that biomimetic hydrogels provide a functional niche capable of augmenting cells’ regenerative potential and enhancing wound healing [15]. In the pre-clinical study conducted by our research group, we also observed synergy between the scaffold and the cells utilised, in this case, harvest from bone marrow. Wounds treated with the scaffold without cells showed better progression than the untreated control group; however, those treated with the cell-seeded scaffold exhibited a significantly superior response. Histological analysis of the group treated with cells and the plasma-based scaffold revealed an organised tissue with a near-normal appearance by day 14, along with a higher collagen density compared to the other groups (control and scaffold alone) [10]. Moreover, in an interesting review, Sukmana and coworkers conclude that scaffolds, matrices, and hydrogels serve as essential substrates that promote cell growth and differentiation, fostering a hydrated and caring microenvironment, which supports the idea that these biomaterials enhance the healing process while minimising scarring [16]. Despite differences in the cell types and scaffolds used, these models share a common conceptual foundation with our approach, which is based on a plasma-derived scaffold combined with bone marrow mononuclear cells. Here, we present the results obtained in the first patient enrolled in the clinical trial “Treatment of difficult-to-heal wounds with autologous bone marrow mononuclear cells (BM-MNCs) embedded in a plasma carrier” (EUCT2023-505017-25-02). This new ATMP combines the beneficial properties of plasma—such as its protein content and natural gel-forming capacity—with those provided by BM-MNCs, including angiogenic effects, immunomodulatory potential, and differentiation capacity, all of which have been extensively described in the literature. BM-MNCs have proven to be safe and effective cells for the treatment of various pathologies. In this context, our group has published studies on the use of BM-MNCs resuspended in a saline solution to treat pressure ulcers and pseudoarthrosis [8,9]. Other authors, such as Liang et al., have utilised these cells in combination with platelet-rich plasma to treat non-traumatic osteonecrosis of the femoral head (ARCO II-IIIA stage), reporting this combination to be safe and effective [17]. In our patient, this treatment led to complete resolution of the ulcer in approximately six weeks. It is noteworthy that this wound had been long-standing and refractory to multiple conventional treatments. This therapeutic approach was feasible, was safe, and provides positive clinical signals, achieving complete healing within a relatively short time frame. The patient’s quality of life improved significantly, with pain reduction evident well before completion of follow-up, and, importantly, no adverse effects were observed. However, certain limitations of the study should be noted; as this is a case report involving a single patient, the results cannot be generalised, which is why although these initial results are promising, further confirmation is required, as this represents the first patient treated with this therapy.

## Data Availability

The original contributions presented in this study are included in the article. Further inquiries can be directed to the corresponding author.
